# Prevalence and factors associated with malaria parasitaemia in children under the age of five years in Malawi: A comparison study of the 2012 and 2014 Malaria Indicator Surveys (MISs)

**DOI:** 10.1371/journal.pone.0175537

**Published:** 2017-04-11

**Authors:** Maggie Zgambo, Balwani Chingatichifwe Mbakaya, Fatch Welcome Kalembo

**Affiliations:** 1 St John’s College of Nursing, Mzuzu, Malawi; 2 Mzuzu University, Private Bag 201, Luwinga Mzuzu 2, Malawi; Institut Pasteur, FRANCE

## Abstract

**Background:**

Malaria is the main cause of morbidity and mortality among children under the age of five years in Malawi. The aim of this study was to compare the prevalence and factors associated with malaria parasitaemia among children under the age of five years in Malawi between the 2014 and 2012 Malaria Indicator Surveys (MISs).

**Methodology:**

Data on demographic factors, vector control interventions, and blood for malaria test were collected from a representative sample of children under the age of five years in Malawi through multistage cluster sampling method. Data were analysed by chi-square test and logistic regression using complex samples analysis of the Statistical Package for the Social Sciences (SPSS) version 22.

**Results:**

The prevalence of malaria parasitaemia among children under the age of five years increased from 28% in 2012 to 33% in 2014 (p > 0.05). Likewise, the proportion of children using long-lasting insecticide-treated net (LLIN) increased significantly from 54% in 2012 to 65% in 2014 MIS (p < 0.05). The proportion of households that had used indoor residual spraying (IRS) was 9% for both surveys. In multivariate analysis, use of LLIN significantly predicted for malaria parasitaemia in the 2012 MIS but not in the 2014 MIS. Older children and those coming from the poorest families were significantly associated with having malaria parasites in both surveys.

**Conclusion:**

The increase in the use of LLIN among children in 2014, did not result in the reduction of malaria parasitaemia in children. The use of LLIN significantly predicted for malaria parasitaemia among children in the 2012 MIS but not in the 2014 MIS. The results of this study underscore the need to increase the coverage of IRS, mosquito repellents and larvicide alongside LLINs in order to reduce the burden of malaria among children in Malawi.

## Introduction

Malaria is a leading cause of mortality among children in sub-Saharan Africa [1]. Globally the estimated number of death due to malaria in 2015 was 438 000, and 91% of them occurred in sub-Saharan Africa [2]. In 2013, malaria was the leading cause of death for children under the age of five years in Malawi, accounting for 22% of all deaths of the under-five children [3]. Malaria is also a major cause of admission for children under the age of five years in Malawi [4]. More than half of all admissions to hospitals for children under the age of five years are due to malaria [5].

Although people from all areas of Malawi are at risk of malaria, those staying in wetter, hotter and more humid, low-lying areas such as along the lakeshore and Shire river valley are at greater risk than those staying in dry and highland areas [4, 6]. In addition, the transmission pattern has a seasonal variation with higher transmission occurring during the rainy season from November to April [7]. The hot and humid weather coupled with the prevalent stagnant water in rainy season provide a favourable environment for the breeding and growth of anopheles mosquitoes, which are responsible for the transmission of malaria parasites [6, 8].

The Government of Malawi and international donors are investing a lot of resources towards interventions to reduce the burden of malaria among children under the age of five years [4, 7]. For example, between 2012 and 2014, the Government of Malawi and its partners distributed 6.7 million free long-lasting insecticides treated nets (ILLNs) to children and pregnant women countrywide [9]. In 2010, the Government of Malawi also rolled out the indoor residual spraying (IRS) in seven districts of the country [4, 7].

Despite the Government of Malawi and its development partners increasing resources each year to reduce the burden of malaria in children under the age of five years, little is known as to whether the interventions are well targeted. Without identifying the predictors of malaria parasitaemia in Malawi, government and its donors might be losing the battle in the fight against malaria despite investing a huge amount of resources. This study aimed to evaluate the effectiveness of malaria interventions in Malawi by comparing the prevalence of malaria and its predictors between the 2014 Malaria Indicator Survey (MIS) and 2012 MIS. Not only will this study provide feedback to government and stakeholders on the effectiveness of malaria control interventions, but will also provide a basis for planning of resources and interventions to control the burden of malaria among under five years old children in Malawi.

## Materials and methods

### Study area

Malawi has a sub-tropical type of climate which favours breeding of anopheles mosquitoes. Breeding of mosquitoes is high in the rainy season, (from November to April) because of the abundance of water, vegetation, and warm weather.

### Sample design

Malawi is divided into three administrative regions (Southern, Central, and Northern) and 28 districts (13 in the Southern Region, nine in the Central and six in the Northern Region). Each district is divided into traditional authorities. For statistical purposes, the 2008 National Population and Housing Census divided these traditional authorities further into standard enumeration areas (SEAs). The 2008 Census also determined the number of households in each area. The 2012 and 2014 Malawi Malaria Indicator Surveys’ sampling frame was the list of SEAs developed from the 2008 Census. The first stage involved stratification of the SEAs by region and residence location (urban and rural). Thereafter 140 SEAs (90 from a rural area and 50 urban area) and 3500 households were selected from the list of 12,569 SEAs from the 2008 census using probability to proportional size. In order to produce robust estimates, the Northern region, and urban areas in the three regions were oversampled making the MIS disproportional to the region and urban population hence needed final weighting adjustment in order to provide reliable estimates for every area of the study. The SEA size was considered as the total number of residential household. The residential households were allocated to the different strata. The second stage involved selection of an average of 25 households from each of the 140 SEAs using systematic sampling from the list of households in the SEAs. Taking into account that it had been several years since the 2008 census was conducted; a fresh household listing was done for both MIS before the commencement of the surveys. New serial numbers were assigned to households during the fresh household listing and were later used to sample participants.

### Data collection and study measures

Data collection for the 2014 MIS were collected from 2 May 2014 to 10 June 2014 while the 2012 MIS data were collected from April to May 2012. The data collection team consisted of community nurses, laboratory technicians, and the supervisors. The 2014 MIS had a response rate of 99% while the 2012 MIS had a response rate of 98%. The 2014 and 2012 MISs used similar study measures namely; the household, the biomarker, and the questionnaire for women. This paper used data from the household and the biomarker questionnaires. The household questionnaire collected information about all members and visitors of the selected households. The purpose of the questionnaire was to identify eligible women for individual interviews and eligible children aged between six and 59 months to be tested for malaria. The instrument also collected demographic characteristics of the study participants such as age, sex, and the relationship of each household member to the head of the family. In addition, the questionnaire collected information pertaining to dwelling characteristics of the dwelling unit such as toilet facility, the source of water, materials used for the wall, floor and roof of the house, ownership of durable goods as well as ownership and use of the mosquito nets. The socioeconomic status of participants was measured using the wealth index. The wealth index, is a tool developed by the World Bank to measure the household socioeconomic status in developing countries on the basis of household’s ownership of consumer goods, dwelling characteristics, type of drinking water source, toilet facilities, and other characteristics [10]. These assets are combined into a composite index of economic status (poorest, poorer. medium, wealthier and wealthiest) using Principal Component Analysis (PCA) [11]. The biomarker questionnaire collected information about malaria test results for children between the ages of six and 59 months.

### Malaria testing

Malaria parasites were detected by microscopy. A thick blood smear was taken from all eligible children and was sent to the laboratory to be microscopically tested for *Plasmodium falciparum*.

### Data analysis

The outcome variable of the study was testing positive or negative for malaria parasites using microscopic test. Malaria results by microscopy were chosen over rapid test because they are more accurate than those by the latter [12]. Given that the latter tests for *Plasmodium falciparum* specific protein rather than the parasite itself hence it is less sensitive than the microscopic test.

The independent variables were divided into two categories; sociodemographic variables and vector control variables. The sociodemographic variables included the following variables; child’s age, sex, mother’s education, residence, region cluster altitude and household wealth index. Vector control variables consisted of ownership of mosquito net, use of LLIN, and use of IRS.

Statistical Package for the Social Sciences (SPSS), IBM version 22 was used to analyse data. MIS used multistage sampling to recruit participants hence it is regarded as having a complex survey design [13]. Analysis of data from complex survey design requires taking into account the sample design in order to prevent bias because of the following reasons: few clusters are sampled hence leaving out a considerable number of clusters unsampled, this may miss out some characteristics in the study. In addition, there might be a correlation of certain characteristics in the same cluster or household hence the assumption of independent test cannot be met. Sometimes, during the selection of study participants from the clusters, unequal weights are used in order to obtain robust estimates, which may result in some areas being oversampled that might lead to the sample being unrepresentative [14]. In light of this explanation, we weighted the data first before descriptive analysis in order to make the sample representative of the general population in Malawi. Pooled data from the two surveys were analysed first to compare malaria vector control indicators between the two surveys and thereafter, the two surveys were analysed separately to identify predictors of malaria parasitaemia. We conducted descriptive statistics in order to describe the characteristics of the study participants. Comparison between categorical variables was conducted by chi-square test. Logistic regression was then used to identify crude odds ratios of factors associated with malaria parasitaemia in children under the age of five years. Variables that were significant at ≤ 0.25 in bivariate analysis (logistic regression) were entered in multiple logistic regression model to identify adjusted odds ratios for factors associated with malaria parasitaemia [15]. The p-value was considered significant at 0.05. All analyses were conducted using complex samples analysis of the Statistical Package for the Social Sciences (SPSS), IBM version 22.

### Ethical clearance

Ethical clearance to conduct the study was obtained from Malawi National Health Science Research Committee (NHSRC) prior to data collection. Verbal and written consent were also obtained from the study participants. Parental consent was also sought from all children who were tested blood for malaria parasites. In addition, the authors of this paper sought permission from the International Classification of Functioning, Disability, and Health (ICF), which oversees demographic health survey data including MIS to publish the results of the study.

## Results

### Prevalence of malaria parasitaemia and general characteristics of the study participants

Overall, a total of 4040 children under the age of five years were included in the study, 2112 (52%) from the 2012 MIS and 1928 (48%) from the 2014 MIS data. The prevalence of malaria parasitaemia increased among children under the age of five years from 28% in 2012 to 33% in 2014. Nevertheless, the difference in prevalence of malaria parasitaemia among children between the two surveys was statistically insignificant (p >0.05). As regards to the proportion of households who owned at least one bed net, the 2014 MIS had a significantly higher proportion (77%) compared to the 2012 MIS (70%) (p <0.05). In addition, the proportion of children who slept under LLIN a night before the survey significantly increased from 60% in 2012 to 66% in 2014 (p <0.05). Furthermore, only nine per cent of households had used IRS in the previous 12 months in both surveys (p > 0.05). When the sociodemographic variables in the two surveys were compared, no statistically significant differences were found in the proportions of participants in any of the variables (wealth index, type of residence, mother’s education, child’s age and child’s sex) between the 2012 MIS and 2014 MIS (p > 0.05) (see Table 1).

**Table 1:** Comparison of vector interventions and sociodemographic indicators between the 2012 and 2014 MISs.

Indicator	2012 and 2014 pooled data estimates N = 4202 N (%)	Comparison of the 2012 and 2014 MIS data				Comparison p—value
		2012 MIS estimates N = 2173 (52%)		2014 MIS estimates N = 2029 (48%)		
		N	% (95% CI)	N	% (95% CI)	
**Vector control**						
Malaria parasitaemia						0.189
Negative	2926 (70)	1571	72 (68–76)	1355	67 (59–74)	
Positive	1276 (30)	602	28 (24–32)	673	33 (26–41)	
Has mosquito bed net for sleeping						0.032
No	1130 (27)	660	30 (36–35)	470	23 (19–28)	
Yes	3072 (73)	1513	70 (65–74)	1559	77 (72–81)	
Child slept under LLIN net last night						0.002
No	1693 (40)	996	46 (41–51)	697	34 (30–40)	
Yes	2509 (60)	1177	54 (49–59)	1332	66 (60–71)	
Dwelling sprayed against mosquitoes						0.976
No/ Do not know	3822 (91)	1975	91 (86–94)	1846	91 (85–95)	
Yes	380 (9)	198	9 (6–14)	183	9 (5–15)	
**Demographic characteristics**						
Region						0.433
Northern	694 (17)	307	14 (11–17)	387	19 (13–28)	
Central	1698 (40)	920	42 (37–47)	778	38 (30–48)	
Southern	1810 (43)	946	44 (39–48)	864	43 (33–53)	
Place of residence						0.795
Urban	558 (13)	281	13 (11–16)	276	14 (10–19)	
Rural	3644 (87)	1892	87 (84–90)	1753	86 (81–90)	
Wealth Index						0.831
Poorest	962 (23)	475	22 (19–25)	486	24 (20–28)	
Poorer	921 (22)	503	23 (20–26)	417	21 (17–25)	
Middle	835 (20)	434	20 (17–23)	402	20 (17–23)	
Richer	808 (19)	413	19 (16–22)	396	19 (16–23)	
Richest	676 (16)	348	16 (13–19)	328	16 (16–23)	
Sex						0.070
Male	2027 (48)	1011	47 (44–49)	1016	50 (47–53)	
Female	2175 (52)	1162	53 (51–56)	1013	50 (47–53)	
Child’s age in months						0.533
6–11	432 (10)	215	10 (9–12)	218	10 (9–13)	
12–23	1016 (24)	509	23 (22–26)	506	25 (22–28)	
24–35	941 (23)	479	22 (20–24)	461	23 (20–26)	
36–47	890 21)	489	23 (21–25)	401	20 (17–23)	
≥48	923 (22)	480	22 (20–24)	443	22 (20–24)	
Mother’s education level						0.557
No education	730 (17)	403	19 (16–22)	327	16 (13–20)	
Primary	2440 (58)	1247	57 (64–60)	1193	59 (55–63)	
Secondary/higher	1032 (25)	523	24 (21–27)	509	25 (21–29)	
Altitude cluster in metres						0.582
≤1000	2208 (53)	1180	54 (46–62)	1028	51 (41–61)	
≥1000	1994 (47)	993	46 (38–54)	1001	49 (39–60)	

All proportions adjusted for study design and sample weight. Percentage rounded up to the nearest whole number

### Factors associated with malaria parasitaemia in bivariate analysis

In bivariate analysis, children from the Central Region, living in rural area, coming from poorest families, older than 11 months, and those whose mothers had lower education level were more likely to have malaria parasites in the 2012 MIS (p < 0.05) (see Table 2). On the other hand, children who had one or more of the following characteristics in the 2014 MIS were more likely to have malaria parasites: coming from a household without a bed net; did not sleep under LLIN a night before the survey; living in rural area; coming from poorest families; older than 47 months; and having a mother who had no formal education (p < 0.05) (see Table 3). In addition, IRS use was significantly not associated with malaria parasitaemia in children in both surveys.

**Table 2:** Factors associated with malaria parasitaemia in 2012 MIS.

Indicator	Total N = 2173 N (%)	Positive N = 602 N (%)	UOR (95%CI)	p-value	AOR (95% CI)	p-value
**Vector control**						
Has mosquito bed net for sleeping				0.164		0.351
No	660 (30)	203 (31)	1.2 (0.9–1.7)		0.9 (0.6–1.2)	
Yes	1513 (70)	399 (26)	1.0		1.0	
Child slept under LLIN net last night				0.133		0.036
No	996 (46)	301 (30)	1.3 (0.9–1.7)		1.4 (1.1–1.9)	
Yes	1177 (54)	301 (26)	1.0		1.0	
Dwelling sprayed against mosquitoes				0.361		
No/ Do not know	1975 (91)	557 (28)	1.3 (0.7–2.3)			
Yes	198 (9)	45 (23)	1.0			
**Demographic characteristics**						
Region				0.032		0.045
Northern	307 (14)	61 (20)	0.7 (0.4–15)		0.8 (0.4–1.5)	
Central	920 (42)	315 (34)	1.7 (1.1–2.6)		1.6 (1.1–2.6)	
Southern	946 (44)	226 (24)	1.0		1.0	
Place of residence				<0.001		0.003
Urban	281 (13)	28 (10)	1.0		1.0	
Rural	1892 (87)	574 (30)	3.9 (2.3–6.9)		2.4 (1.4–4.2)	
Wealth Index				<0.001		0.004
Poorest	475 (22)	179 (38)	5.3 (3.0–9.5)		2.9 (1.6–5.3)	
Poorer	503 (23)	154 (31)	3.9 (2.0–7.6		2.3 (1.1–4.6)	
Middle	434 (20)	135 (31)	4.0 (2.2–7.3)		2.5 (1.3–5.0)	
Richer	413 (19)	100 (24)	2.8 (1.6–5.0)		1.9 (1.1–3.6)	
Richest	348 (16)	34 (10)	1.0		1.0	
Sex				0.516		
Male	1011 (47)	273 (27)	0.9 (0.8–1.1)			
Female	1162 (53)	329 (28)	1.0			
Child’s age in months				<0.001		<0.001
6–11	215 (10)	42 (7)	1.0		1.0	
12–23	509 (23)	107 (18)	1.1 (0.7–1.8)		1.1 (0.7–1.8)	
24–35	479 (22)	148 (24)	1.9 (1.2–3.0)		2.0 (1.2–3.4)	
36–47	489 (23)	147 (24)	1.8 (1.2–2.7)		1.8 (1.2–2.9)	
≥48	481 (22)	158(33)	2.0 (1.3–3.1)		2.1 (1.3–3.3)	
Mother’s education level				<0.001		0.045
No education	403 (19)	137 (34)	2.2 (1.5–3.1)		1.5 (1.1–2.2)	
Primary	1247 (57)	364 (29)	1.7 (1.3–2.3)		1.4 (1.1–2.0)	
Secondary/higher	523 (24)	101 (19)	1.0		1.0	
Altitude cluster in metres				<0.587		
≤1000	1180 (54)	314 (27)	0.9 (0.6–1.4)			
≥1000	993 (46)	288 (29)	1.0			

All proportions adjusted for study design and sample weight. Percentage rounded up to the nearest whole number

**Table 3:** Factors associated with malaria parasitaemia in 2014 MIS.

Indicator	Total N = 2029 N (%)	Positive N = 673 N (%)	UOR (95%CI)	p-value	AOR (95% CI)	p-value
**Vector control**						
Has mosquito bed net for sleeping				0.024		0.776
No	470 (23)	191 (41)	1.5 (1.1–2.2)		0.9 (0.5–1.7)	
Yes	1559 (77)	482 (31)	1.0		1.0	
Child slept under LLIN net last night				0.018		0.146
No	698 (34)	271 (39)	1.5 (1.1–2.0)		1.5 (0.9–2.4)	
Yes	1331 (66)	402 (30)	1.0		1.0	
Dwelling sprayed against mosquitoes				0.161		0.225
No/ Do not know	1846 (91)	584 (32)	0.5 (0.2–1.3)		0.5 (0.2–1.5)	
Yes	183 (9)	89 (49)	1.0		1.0	
**Demographic characteristics**						
Region				0.573		
Northern	387 (19)	110 (28)	0.8 (0.3–2.0)			
Central	778 (38)	283 (36)	1.1 (0.6–2.5)			
Southern	864 (43)	280 (32)	1.0			
Place of residence				<0.001		0.075
Urban	276 (14)	30 (11)	1.0		1.0	
Rural	1753 (86)	643 (37)	4.8 (2.4–9.5)		2.3 (0.9–6.0)	
Wealth Index				<0.001		0.001
Poorest	486 (24)	237 (49)	7.3 (2.9–18.8)		4.7 (1.3–16.2)	
Poorer	417 (21)	155 (37)	4.5 (1.9–11.0)		2.9 (0.9–10.0)	
Middle	402 (20)	141 (35)	4.2 (1.5–11.9)		2.7 (0.7–10.2)	
Richer	396 (19)	103 (26)	2.7 (0.9–8.4)		1.9 (0.4–8.0)	
Richest	328 (16)	37 (12)	1.0		1.0	
Sex				0.340		
Male	1016 (50)	350 (35)	1.1 (0.9–1.5)			
Female	1013 (50)	323 (32)	1.0			
Child’s age in months				<0.001		<0.001
6–11	217 (10)	55 (25)	1.0		1.0	
12–23	506 (25)	120 (24)	0.9 (0.6–1.5)		0.8 (0.5–1.4)	
24–35	461 (23)	165 (36)	1.6 (1.0–2.6)		1.5 (1.0–2.5)	
36–47	402 (20)	151 (38)	1.8 (1.0–3.1)		1.6 (0.9–2.9)	
≥48	443 (22)	182 (41)	2.1 (1.3–3.2)		2.2 (1.4–3.5)	
Mother’s education level				0.004		0.392
No education	327 (16)	137 (42)	2.1 (1.4–3.3)		1.3 (0.8–2.1)	
Primary	1194 (59)	408 (34)	1.5 (1.0–2.3)		1.0 (0.6–1.6)	
Secondary/higher	508 (25)	128 (25)	1.0		1.0	
Altitude cluster in metres				0.289		
≤1000	1028 (51)	378 (37)	1.4 (0.8–2.6)			
≥1000	1001 (49)	295 (30)	1.0			

All proportions adjusted for study design and sample weight. Percentage rounded up to the nearest whole number

### Factors associated with malaria parasitaemia in multivariate statistics

The odds of testing positive for malaria parasites in multivariate analysis in the 2012 MIS were higher among children who did not sleep under the LLIN the night before the survey (AOR 1.4; 95% CI: 1.1–1.9), children from the Central Region (AOR 1.6; 95% CI: 1.1–2.6), children living in rural area (AOR 2.4; 95% CI: 1.4–4.2), children from poorest families (AOR 5.3; 95% CI: 3.0–9.5), children who were 48 months of age or older (AOR 2.1; 95% CI: 1.3–3.3) and mothers of children with no education (AOR 1.5; 95% CI: 1.1–2.2) (see Table 2). On the other hand, two variables namely, child’s age and wealth index were identified as significant predictors of malaria parasites in the 2014 MIS. Just like in the 2012 MIS, children from poorest families in the 2014 MIS were 4.7 times more likely to test positive for malaria parasites compared to those from the richest families. In terms of age, the odds of testing positive for malaria parasites were higher in children who were 48 months of age or older compared to those who were younger (AOR 2.2: 95% CI 1.4–3.5) (see Table 3).

## Discussion

The results of this study have shown that the prevalence of malaria parasitaemia among children younger than five years was higher in 2014 compared to 2012 despite a significant increase in the use of LLIN. The findings of this study are not different from those reported in a previous cohort study conducted in Uganda among 100 children who were followed up from six weeks of age until they were 48 months of age [16]. In the Ugandan study, all children were provided with LLINs and the compliance to the use of LLINs was 98 percent, however, despite the high rate of compliance to LLIN use, the incidence of malaria increased by 52% by the end of the follow-up time, moreover, all but five children were diagnosed with malaria within the follow-up period. Authors of studies in sub-Saharan countries have also reported a lack of reduction in malaria parasitaemia after an increase in the number of insecticide-treated nets [17, 18]. Conversely, some studies have reported a reduction in malaria parasites after increasing the coverage of LLIN [19, 20].

In addition, this study has found that not sleeping under LLIN the night preceding the survey was a significant predictor of malaria parasites in children under the age of five years in the 2012 MIS but not in the 2014 MIS. Authors of previous studies have also reported different findings regarding LLIN, with some identifying it as a significant predictor of malaria parasites among children [21, 22] and others report it as not significantly associated with malaria parasites in children under the age of five years [12, 23]. The difference between the two surveys regarding LLIN as a predictor of malaria parasitaemia may be attributed to a number of factors. First, development of resistance to pyrethroids (an insecticide used in LLIN) in mosquitoes in 2014 might be a possible explanation considering that resistance to pyrethroids used in IRS, has already been identified in mosquitoes in Malawi [24]. Another possible factor for the difference might be underutilisation of LLINs in 2014 considering that recently there have been reports in Malawi regarding LLINs being used to harvest fish in Lake Malawi [25]. Another reason might be primary caregiver’s lack of knowledge on malaria in 2014. A previous study found that lack of knowledge on malaria affects compliance to LLIN use [26]. This study did not assess the association between knowledge on malaria and utilisation of LLIN in Malawi. Future studies should consider assessing the relationship between these two factors.

This study has also shown that although the use of LLIN increased in 2014, the proportion of households that used IRS did not increase between 2012 and 2014. In 2010, the Government of Malawi and its donors recognising the importance of vector control, initiated the distribution of LLINs and indoors residual spraying (IRS) with pyrethroid insecticide [27]. By 2011 the IRS programme had covered seven of the malaria endemic districts in Malawi [24]. However, resistance to pyrethroids was identified in mosquitoes in some of the districts initially covered by the IRS programme[24]. This necessitated the change from pyrethroids IRS to a short acting but expensive insecticide, organophosphate [28], which made the IRS programme more expensive. Consequently, the President’s Malaria Initiative (PMI), a US-funded organisation, which was the main donor for IRS, withdrew funding for the IRS because it was becoming expensive [24]. While the government increased the countrywide coverage of LLINs, it failed to scale up the IRS programme beyond the initial seven districts due to lack of funds [28]. This provides an explanation to the increase in the proportion of children who slept under LLIN a night prior to the survey in 2014 and the lack of increase in the proportion of households which had used IRS in the 2014 MIS. Literature has shown that supplementing the use of LLIN with IRS helps to reduce malaria parasitaemia[29]. For example, Botswana is one of the few countries in sub-Saharan Africa that has made a significant progress in reducing the prevalence of malaria parasites in children under the age of five years and is close to eliminating malaria through scaling up and sustained provision of both LLIN and IRS [29]. The Government of Botswana has managed to reduce the prevalence of malaria by 98% through the use of IRS and LLINs within a period of five years [29].

In both the 2012 MIS and 2014 MIS, children from poor families were more likely to suffer from malaria compared to those from wealthiest families. There is a great deal of literature, which shows that poverty is a driver of malaria [1, 30–32]. Poor people tend to reside in houses that expose them to mosquito bite [33]. According to the findings of two studies conducted in Rwanda and Uganda children from the higher socioeconomic status families were more likely to sleep under insecticide-treated nets compared to those from lower socioeconomic status [34, 35]. The findings of this study are similar to those of a MIS conducted in Gambia, which reported that children from the poorest quintiles were more likely to suffer from malaria as compared to those from richest quintiles [36].

Furthermore, this study has revealed that older children under the age of five years were more likely to test positive for malaria parasites compared to younger children in both the 2012 and 2014 surveys. Normally, the child’s care pattern in Malawi is that younger children (6 to 11 month) are given more priority and are in the total care of their mothers or guardian whereas older children spend more time in the evening playing with siblings before going to bed, and in the process they are bitten by mosquitoes. At night they may move close to the edge of the net and are susceptible to mosquito bites. Younger children in Malawi normally share the same bed with their mothers as such they are properly covered under the mosquito net at night. A study conducted in Uganda showed that children who share a bed with their mother were 21 times more likely to use the mosquito nets compared to those who were not [35]. The finding reported in this study is similar to that from previous studies which reported that malaria infection in children under the age of five years increased with age [12, 22, 37].

### Implications for practice

The results of this study highlight the need for increased efforts to reduce the burden of malaria among children under the age of five years in Malawi. Increasing the coverage of long-acting organophosphate IRS should be one of the government’s priority vector control intervention. IRS can have significant results in the fight against malaria as children can be protected from mosquito bites outside bedtime. A study conducted in Uganda using the MIS data found that the odds of the suffering from malaria were 7.6 times in houses not sprayed against mosquitos as compared to those who were living in houses sprayed against mosquitoes [12]. Moreover, countries that have made significant progress in controlling malaria in children under the age of five years have increased the coverage of both LLIN and IRS.

Importantly, considering that children spend a larger part of the day playing outdoors, introducing government or donor-subsidized mosquito repellents can help to protect children from mosquito bites. In rainy season many houses in Malawi, especially in rural areas are surrounded by stagnant water, which provides a potential breeding site for mosquitoes. Larviciding stagnant water around peoples' houses can help to control the population of mosquitoes and so reduce the burden of malaria parasitaemia in Malawi. The results of this study could inform health workers to develop a relevant intervention to address the predictors of malaria among under-five children. When providing health education to primary caregivers, emphasis should be placed on encouraging primary caregivers to ensure that both younger and older children under the age of five years sleep under the LLINs every night. Furthermore, when coming up with policies, guidelines, and interventions to control malaria in children under the age of five years, special attention should be paid to older children and those from poor families.

### Strengths and limitations of the study

The main strengths of this study are twofold; first, this study used data from two national surveys that had a high response rate of greater than 95%. In addition, the study used survey data collected from a national representative sample as such, the findings can be generalised to children under the age of five years in Malawi. Just like many studies, this study also has limitations. First, the study used cross-sectional design to collect data as such no causal inferences can be made. The second limitation is that we did not include any meteorological data to assess if the climatic variations contributed to the difference in prevalence of malaria parasitaemia. Another limitation of the study is that we did not take into account the seasonal variation in malaria transmission pattern between 2012 and 2014 taking into consideration that the 2014 survey was conducted a month later than the 2012 survey. Future studies should consider assessing the association of climatic variations and malaria parasitaemia in children. Despite these limitations, this study has identified some of the determinants of malaria parasitaemia in children under the age of five years in Malawi that can be used for planning future interventions.

## Conclusion

The comparison of the two surveys shows that little progress has been made in reducing malaria parasitaemia among children under the age of five years. The increase in the proportion of children who slept under LLIN a night preceding the survey in 2014, did not result in a reduction of malaria parasitaemia in children. Moreover, the proportion of households that used IRS did not increase in 2014. Socio-demographic factors such as older age and coming from poor families were independent predictors of malaria parasitaemia for the two surveys. The results of this study show that use of LLIN alone is not enough to reduce malaria parasitaemia in children in Malawi. The Government of Malawi and international donors should consider increasing the coverage of other interventions such as IRS, mosquito repellent and Larviciding in order to reduce the burden of malaria parasitaemia among children under the age of five years in Malawi. In addition, it is important to take into consideration the socio-demographic factors that are associated with malaria parasites among children in Malawi when planning or implementing vector control interventions in order to have positive outcomes. This study has created a benchmark for the planning of malaria control interventions as well as for comparing progress and successes of future interventions in Malawi.
